# Gnathostomosis in fish from Tres Palos Lagoon, Guerrero, Mexico.

**DOI:** 10.3201/eid0604.000424

**Published:** 2000

**Authors:** V León-Règagnon, L García-Prieto, D Osorio-Sarabia, A Jiménez-Ruiz


sporadic serogroup C ET-24 strain. The 16S rRNA gene sequences of 66 *N. meningitidis* isolates representing serogroups A, B, C, W135, Z, and Y were diverse, with nine different sequences among the NMW135 isolates. Finally, all four recent NMW135 isolates had identical *Nhe*I pulsed-field gel electrophoresis (PFGE) patterns distinct from patterns seen in other NMW135 isolates. All these molecular markers were clearly unique in NMW135 isolates previously identified in the United States or isolated at the same time as the Hajj-associated isolates but with no epidemiologic link to the current outbreak. These unique markers allowed easy differentiation of the imported, Hajj-associated isolates from other sporadic NMW135 isolates circulating in the United States.

It has been shown previously that NMW135 strains can exist in widely divergent clonal groups. Our data suggest that strains like those associated with this year's Hajj have been in circulation in human populations for at least several years in different parts of the world. Given that the Hajj is a large, yearly event, high-level exposure of pilgrims to respiratory secretions and subsequent spread of infection to many countries by returning pilgrims may turn W135 meningococcal disease into a global health threat. Continued surveillance, as well as increased awareness of meningococcal disease caused by *N. meningitidis* of this serogroup by physicians and the public, is needed. Efforts to measure the efficacy of the quadrivalent meningococcal vaccine for prevention of W135 meningococcal disease should be considered. To get a better global understanding of W135 meningococcal disease, we are conducting a large multicenter study on molecular characterization of >50 Hajj-associated NMW135 isolates from Saudi Arabia, France, Singapore, and Finland, and 50 other W135 isolates from throughout the world.

**Tanja Popovic,* C.T. Sacchi,* M.W. Reeves,* A.M. Whitney,* L.W. Mayer,* C.A. Noble,* G.W. Ajello,* F. Mostashari,*† N. Bendana,‡ J. Lingappa,* Rana Hajjeh,* and N.E. Rosenstein***

*Centers for Disease Control and Prevention, Atlanta, Georgia, USA; †New York City Department of Health, New York, New York, USA; and ‡California Department of Health, Los Angeles, California, USA

### References

1. Samuelsson S, Handysides S, Ramsay M, Lyytikainen O, Nygard K, Perrocheau A, et al. Meningococcal infection in pilgrims returning from the Haj: update from Europe and beyond. Eurosurveillance Weekly 2000:17:1-5.
2. Achtman M. Molecular epidemiology of epidemic bacterial meningitis. Rev Med Microbiol 1990:1:29-38.
3. Novelli VM, Lewis RG, Dawood ST. Epidemic group A meningococcal disease in Haj pilgrims. Lancet 1987:2:863.
4. Centers for Disease Control and Prevention. Serogroup W135 meningococcal disease among travelers returning from Saudi Arabia—United States, 2000. MMWR Morb Mortal Wkly Rep 2000:49:345-6.
5. Popovic T, Ajello G, Facklam R. Laboratory manual for the diagnosis of meningitis caused by *Neisseria meningitidis, Streptococcus pneumoniae,* and *Haemophilus influenzae.* Geneva: World Health Organization; 1999.
6. Caugant D. Population genetics and molecular epidemiology of *Neisseria meningitidis.* APMIS 1998:106:505-25.
7. Rosenstein NE, Perkins BA, Stephens D, Lefkowitz L, Cartter ML, Danila R, et al. The changing epidemiology of meningococcal disease in the United States, 1992-1996. J Infect Dis 1999:180:1894-901.
8. Reeves MW, Perkins BA, Diermayer M, Wenger JD. Epidemic-associated *Neisseria meningitidis* detected by multilocus enzyme electrophoresis. Emerg Infect Dis 1995:1:53-4.
9. Sacchi CT, Lemos APS, Brandt ME, Whitney AM, Melles CAE, Solari CA, et al. Proposed standardization of *Neisseria meningitidis* PorA variable-region typing nomenclature. Clin Diagn Lab Immunol 1998:5:845-55.
10. Kwara A, Adegbola RA, Corrah PT, Weber M, Achtman M, Morelli G, et al. Meningitis caused by a serogroup W135 clone of the ET-37 complex of *Neisseria meningitidis* in West Africa. Trop Med Int Health 1998:3:742-6.
11. Maiden MCJ, Bygraves JA, Feil E, Morelli G, Russell JE, Urwin R, et al. Multilocus sequence typing: a portable approach to the identification of clones within populations of pathogenic microorganisms. Proc Natl Acad Sci 1998:95:3140-5.

### Gnathostomosis in Fish from Tres Palos Lagoon, Guerrero, Mexico

**To the Editor:** Since the first two cases of human gnathostomosis in Mexico were reported in 1970 (1), >1,500 cases have been reported in Nayarit, Sinaloa, Oaxaca, Guerrero, Veracruz, and Tamaulipas states (2). In Acapulco, Guerrero, 98 cases have been described; the intermediate or definitive hosts in this region are unknown (3,4).

During a survey of parasitic helminths of wild vertebrates from Tres Palos Lagoon, in Guerrero, Mexico, we found *Gnathostoma* sp. advanced third-stage larvae ($AdvL_3$) in the skeletal muscle of several fish species. Fish were caught from March to August 1999 in Tres Palos Lagoon (16° 41' to 16° 50'N and 99° 37' to 99° 47'W), Acapulco Municipality, 25 km south of Acapulco Bay (5). Fish muscle was ground individually, compressed between glass plates, and examined with a magnifying glass and a lamp. The infection was characterized as by Margolis et al. (6).

Of nine fish species examined, five were positive for *Gnathostoma* $AdvL_3$: Eleotridae: *Dormitator latifrons* ("popoyote," n = 83), *Gobiomorus maculatus* ("guavina," n = 66), *Eleotris pictus* ("alahuate," n = 22); Cichlidae: *Cichlasoma trimaculatum* ("charra," n = 62), and Ariidae: *Cathorops caerulescens* ("cuatete," n = 62). The highest prevalence and mean abundance values (number of larvae per fish) were found in *E. pictus* (31.81%, 0.82 ± 1.99); in the other host species values were $\leq$7.22 and 0.072 ± 0.26, respectively. *E. pictus* mean abundance values differed significantly from those of the other host species (nonparametric Kruskal-Wallis test, H = 27.125, 4 g.l., n = 337, p <0.0).

The intermediate host transmitting the infection to humans in Mexico had previously been identified only in the Rio Papaloapan Basin, in Veracruz and Oaxaca (7,8). The presence of *Gnathostoma* $AdvL_3$ in the muscle of fish species frequently eaten by humans in Acapulco suggests that these fish may have been the main source of infection in the 98 recorded cases of gnathostomosis (3,4). The popularity of "ceviche" (raw fish marinated in lime juice), prepared with the most commonly caught fish (including the three species of eleotrids studied), strongly supports this possibility. The identification of the source of human infection allows local health authorities to implement public information campaigns about the risk of eating raw or undercooked fish (in the form of sushi or ceviche) in this region. After this initial step in the study of this parasitic disease, the worm species must be accurately identified. In addition, understanding the parasite's life cycle is important for control of a parasitic disease.

### Acknowledgments

We thank F. Bertoni, E. Martínez, C. Castilla, A. Monet, E. Cabrera, and B. Mendoza for their help in field collections.

This study was supported by project PAPIIT-UNAM IN219198 to VL-R.

**Virginia León-Règagnon, Luis García-Prieto, David Osorio-Sarabia, and Agustín Jiménez-Ruiz**
Universidad Nacional Autónoma de México, México D.F., México

### References

1. Peláez D, Pérez-Reyes R. Gnatostomiasis humana en América. Rev Latinoam Microbiol 1970;12:83-91.
2. Lamothe AR, Osorio SD. Estado actual de la gnatostomiasis en México. Anales del Instituto de Biología de la Universidad Nacional Autónoma de México. Serie Zoología 1998;69:23-37.
3. Rojas-Molina N, Pedraza-Sánchez S, Torres-Bibiano B, Meza-Martínez H, Escobar-Gutiérrez A. Gnathostomosis, an emerging disease in Acapulco, Mexico. Emerg Infect Dis 1999;5:264-6.
4. Vargas OF, Alarcón RE, Alvarado AFJ. Human gnathostomiasis in Mexico. Int J Dermatol 1998;37:433-53.
5. Diego P, Lozada L. Estudios florísticos de Guerrero No. 3. Laguna de Tres Palos. Facultad de Ciencias, Universidad Nacional Autónoma de México; México, D.F., 1994. p. 29.
6. Margolis L, Esch GW, Holmes JC, Kuris AM, Schad GA. The use of ecological terms in parasitology (Report of an ad hoc committee of the American Society of Parasitologists). J Parasitol 1982;68:131-3.
7. Almeyda AJ. Hallazgo de *Gnathostoma binucleatum* n. sp. (Nematoda: Spirurida) en felinos silvestres y el papel de peces dulceacuícolas y oligohalinos como vectores de la gnathostomiasis humana en la cuenca baja del Río Papaloapan, Oaxaca-Veracruz, México. Anales del Instituto de Ciencias del Mar y Limnología, Universidad Nacional Autónoma de México 1991;18:137-55.
8. Lamothe-Argumedo R, Medina-Vences R, López-Jiménez S, García-Prieto L. Hallazgo de la forma infectiva de *Gnathostoma* sp., en peces de Temascal, Oaxaca, México. Anales del Instituto de Biología de la Universidad Nacional Autónoma de México. Serie Zoología 1989;60:311-20.

### First Report of Human Granulocytic Ehrlichiosis from Southern Europe (Spain)

**To the Editor:** Human granulocytic ehrlichiosis (HGE) is a tickborne zoonosis described in the United States several years ago (1) and in Europe recently (2). Several hundred cases

